# Technical-assistance arrangements in coping with the COVID-19 pandemic from the managers’ perspective ^*^

**DOI:** 10.1590/1518-8345.5799.3539

**Published:** 2022-07-08

**Authors:** Kássia Janara Veras Lima, Marcus Vinícius Guimarães de Lacerda, Wagner Ferreira Monteiro, Darlisom Sousa Ferreira, Lucas Lorran Costa de Andrade, Flávia Regina Souza Ramos

**Affiliations:** 1 Universidade do Estado do Amazonas, Escola Superior de Ciências da Saúde, Manaus, AM, Brasil.; 2 Bolsista da Coordenação de Aperfeiçoamento de Pessoal de Nível Superior (CAPES), Brasil.; 3 Fundação de Medicina Tropical Doutor Heitor Vieira Dourado, Manaus, AM, Brasil.; 4 Bolsista do Conselho Nacional de Desenvolvimento Científico e Tecnológico (CNPq), Brasil.; 5 Centro Universitário Luterano de Manaus, Manaus, AM, Brasil.; 6 Bolsista da Fundação de Amparo à Pesquisa do Estado do Amazonas (FAPEAM), Brasil.

**Keywords:** Pandemics, COVID-19, Public Health, Health Management, Health Services, Health Manager, Pandemias, COVID-19, Saúde Pública, Gestão em Saúde, Serviços de Saúde, Gestor de Saúde, Pandemias, COVID-19, Salud Pública, Gestión en Salud, Servicios de Salud, Gestor de Salud

## Abstract

**Objective:**

to describe the technical-assistance arrangements developed within the scope of work management in the COVID-19 pandemic care network, from the managers’ perspective.

**Method:**

a qualitative research study, of the incorporated single case type, conducted with 23 managers of a Health Care Network. The analysis was applied in two thematic coding cycles, with the aid of the ATLAS.ti software.

**Results:**

the arrangements were analyzed in categories related to health care; management; incorporation of technologies; implementation of a field hospital; and retrospective analysis of the experiences as a whole. There was emphasis on the implementation of care flows, virtual health bulletins, Telemonitoring, chatbots, use of applications, and implementation of field hospitals and of basic urgency services within the scope of the Basic Health Units. Hyperjudicialization in the system was identified; as well as weaknesses in information management, intersectoriality and technical-political leadership at the national level; the role of nurses in management positions and for coping with the pandemic.

**Conclusion:**

despite the health services’ unpreparedness to face the pandemic, the actors’ resilience promoted dynamism and technical-assistance arrangements in the context of management and humanized care. The study has a potential to contribute to the qualification of the public policy management and development practices.

## Resumo

**Objetivo:**

descrever os arranjos tecnoassistenciais desenvolvidos no âmbito da gestão do trabalho na rede de atenção à pandemia de COVID-19, na perspectiva de gestores.

**Método:**

pesquisa qualitativa, do tipo caso único incorporado, com 23 gestores de uma Rede de Atenção à Saúde. Análise aplicada em dois ciclos de codificação temática, com o auxílio do *software* ATLAS.ti.

**Resultados:**

os arranjos foram analisados em categorias vinculadas à: atenção à saúde; gestão; incorporação de tecnologias; implantação de hospital de campanha; e análise retrospectiva das experiências como um todo. Houve destaque para a implantação de fluxo de atendimentos, boletins de saúde virtuais, telemonitoramento, *chatbots*, uso de aplicativos, implantação de hospitais de campanha e da urgência básica no âmbito de Unidades Básicas de Saúde. Identificou-se a hiperjudicialização no sistema; fragilidades na gestão das informações, intersetorialidade e condução técnico-política em âmbito nacional; o protagonismo dos enfermeiros em cargos de gestão e para o enfrentamento da pandemia.

**Conclusão:**

apesar do despreparo dos serviços de saúde para o enfrentamento da pandemia, a resiliência dos atores promoveu dinamicidade e arranjos tecnoassistenciais no âmbito da gestão e do cuidado humanizado. O estudo tem potencial contribuição para qualificação das práticas de gestão e desenvolvimento de políticas públicas.

## Resumen

**Objetivo:**

describir los preparativos tecnoasistenciales que se desarrollaron en el ámbito de la gestión del trabajo en la red de atención de la pandemia de COVID-19, desde la perspectiva de los gestores.

**Método:**

investigación cualitativa, del tipo caso único incorporado, con 23 gestores de una Red de Atención de Salud. Análisis aplicado en dos ciclos de codificación temática, con ayuda del *software* ATLAS.ti.

**Resultados:**

los preparativos fueron analizados en categorías relacionadas con: la atención de la salud; la administración; la incorporación de tecnologías; la implementación de un hospital de campaña; y el análisis retrospectivo de las experiencias en general. Se destacaron la implementación del flujo de atención, los boletines virtuales de salud, el telemonitoreo, los *chatbots*, el uso de aplicaciones, la implementación de hospitales de campaña y emergencias básicas en el ámbito de las Unidades Básicas de Salud. Se identificaron la hiperjudicialización en el sistema; las debilidades en la gestión de la información, la intersectorialidad y conducción técnico-política a nivel nacional; el protagonismo de los enfermeros en cargos de gestión y para hacer frente a la pandemia.

**Conclusión:**

a pesar de la falta de preparación de los servicios de salud para enfrentar la pandemia, la resiliencia de los actores promovió el dinamismo y los preparativos tecnoasistenciales en el ámbito de la gestión y de la atención humanizada. El estudio tiene una contribución potencial para la calificación de las prácticas de gestión y el desarrollo de políticas públicas.

**Highlights:**

(1) Organization modalities for the Health Care Network given the public health emergencies.

(2) Development of public policies in the area.

(3) Contribution of Nursing in managing the public services.

(4) Qualification and strengthening of public management practices in a crisis scenario.

(5) Reflections on the technical-political leadership in Brazil and impact on the pandemic.

## Introduction

The COVID-19 pandemic has marked social life since 2020, constituting one of the greatest challenges for public health of this century. Insufficient scientific knowledge about the new virus and its high dissemination and lethality produce fear and insecurity regarding the decisions and strategies to face the epidemic. In Brazil, the situation is aggravated due to social and demographic inequality, as well as to distribution and access to the health services, especially those of greater complexity, in addition to the significant concentration of vulnerable population and high prevalence of chronic diseases^1^.

As a result of the pandemic, drastic changes in social life have been taking place since 2020, and one of the major problems of the present and the future - living - is subordinate to facing the pandemic and the social becoming refers to the “becoming pandemic”^2^. As a consequence, the future of the Unified Health System (*Sistema Único de Saúde*, SUS) emerges not only for its role in combating the pandemic, but as an aggregating element of approximation of different actors around the claim on its strengthening^2^. However, the trap of emergency perspectives and ephemeral solutions must be avoided.

This has challenged health professionals, the scientific community, health managers and rulers who seek to carry out the planning of assertive and rapid assistance, aiming to mitigate contagion and avoid exhaustion of the health systems, in addition to maintaining safe, timely and quality access to the health services^3^.

The pandemic exposed chronic weaknesses of the SUS, experienced by managers and workers. In Amazonas, gaps were aggravated by the demographic and territorial particularities, with a capital city that concentrates nearly 53% of the state’s population and high-complexity services^4^.

Since the first case, on March 19^th^, 2020, Manaus faced two peaks of the disease, becoming the epicenter in the country, in April 2020 and January 2021. In this latter, there was an abrupt increase in the number of cases and deaths that caused made the public and private health services collapse, in addition to the oxygen crisis^5^. On September 24^th^, 2020, Manaus reached nearly 204,266 confirmed cases and 9,464 deaths due to COVID-19^6^.

The impact of the pandemic on the quality of life of individuals and communities has worldwide revealed the importance of developing specific policies and operational guidelines for the crisis context, the need for professionals prepared to promote holistic clinical management of the disease^7^. It requires the application of normative and legal measures to contain COVID-19, aiming at a timely response to the needs of the territory, from the perspective of integrated, accessible and resolute services. Health systems require adjustments in real time, led by the services (strategic/tactical/operational level), resulting in new technical-assistance arrangements^8^.

These arrangements express the logic of the technical-assistance model adopted by the territory, that is, the way win which health care actions are organized, involving scientific and care aspects, and articulation between physical, technological and human resources available to face the health problems of a community^9^.

The use of the concept of technical-assistance arrangement in this study is due to its property of articulating meanings with a theoretical and empirical basis: a commitment to the appreciation of experiences built and captured by concrete subjects (managers) and to what they reveal in rapid transformation or response movements. The arrangements can represent such responses in their developments of technologies and innovations to organize and operate health practices; in short, what is arranged are knowledge and ways of acting on health situations that, in the case of the pandemic, break away with logics and routines and challenge the operating capacities in the health services. For this reason, this concept can be applied to different contexts, helping to capture particularities of local constructions and subjects. 

Specifically regarding the scenario under study, the justification of the research combines the importance of disseminating the experiences of managers with privileged positions and the particularity of the exemplary reality from a national and international point of view, due to the severity of the crisis experienced. In addition to that, the state of the art on this object - of a pandemic still in progress - expresses a large and recent derived scientific production, although still without significant publication on the changes developed with a focus on the management of local services, which still need to be reported.

Reflecting on the functioning of the health services is a valuable opportunity for more critical appropriations of the transformations mobilized by the situations experienced, such as the possible developments for management in health emergencies and the strengthening of public systems and professional performance, in the case of Nursing. Based on this potential, the study was guided by the following question: Which technical-assistance arrangements were devised in the management of the health services to face the COVID-19 pandemic in Manaus? The study aimed at describing the technical-assistance arrangements developed within the scope of work management in the COVID-19 pandemic care network, from the managers’ perspective.

## Method

### Type of study

This is a qualitative research study, outlined as an incorporated single case study^10^. Adequacy of the research design was due to its delimitation to a complex phenomenon (facing the pandemic), in a specific context (municipality), but which required the incorporation of different analysis units (different types of services and management levels) and sources of evidence (interviews with different informants and documents).

The procedures were reported according to the COREQ (Consolidated Criteria for Reporting Qualitative Research) guide guidelines.

### Research scenario

The study was developed in Manaus, capital city of Amazonas, with 2,219,580 inhabitants, representing 52.75% of the state’s population, 13.01% of the Northern Region’s and 1.04% of Brazil’s, being the seventh most populous capital^4^.

The State Health Secretariat of Amazonas (*Secretaria de Estado de Saúde do Amazonas*, SES) is responsible for the formulation and development of policies, aimed at organization of the SUS in Amazonas, running the medium- and high-complexity services in Manaus, with 58 Health Care Institutions (HCIs)^11^. The management of Primary Health Care (PHC), under the coordination of the Municipal Health Secretariat of Manaus (*Secretaria Municipal de Saúde de Manaus*, SEMSA), manages 305 HCIs, representing 67.28% of primary care coverage^4^ ^,^ ^12^.

To fight against COVID-19, the Health Care Network (HCN) was reorganized, having the following priority entry doors: Basic Health Units (BHUs), Emergency Care Services (ECSs), Emergency Care Units (ECUs) and First Aid (FA)^11^ ^-^ ^12^. Clinical hospitalization or in the Intensive Care Unit (ICU) occurs by medical regulation, via the Regulated Emergency Transfer System (*Sistema de Transferência de Emergência Reguladas*, SISTER) and transportation by the Mobile Emergency Care Service (*Serviço de Atendimento Móvel de Urgência*, SAMU)^12^.

### Data collection procedures and instruments

**Figure 1:**
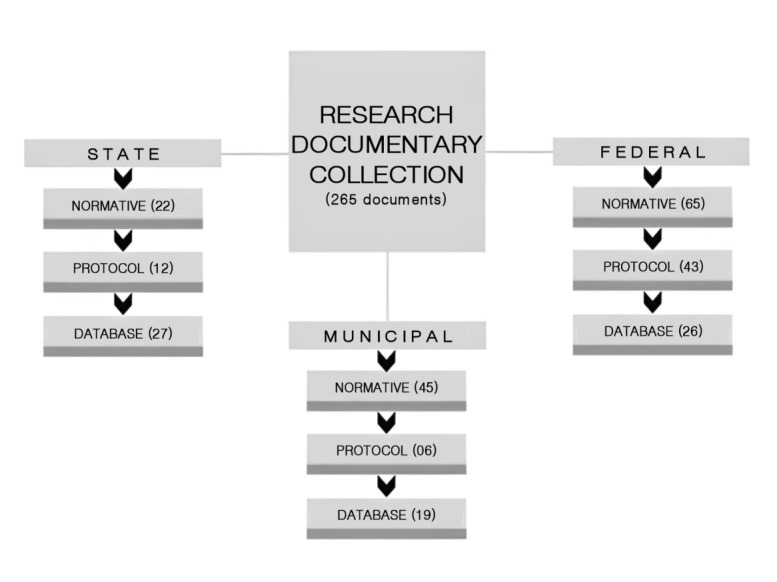
Description of the research documentary collection

The study was conducted in two stages: documentary analysis and interviews. The documentary research was carried out between April and June 2021, covering free access documents available in the institutional websites, of national, state or municipal scope, organized in a specific *Google Drive* and totaling 265 documents (Figure 1). They were systematized in normative, protocol and information systems data, through an Excel spreadsheet prepared by the authors, which contained indication of authorship, date, purpose/subject matter, added to the analytical process for comparison and deepening of the interpretations produced.

The interviews took place from June 22^nd^ to November 10^th^, 2020, carried out by the main author (Master’s degree) and the second (Master’s degree) and fourth (PhD) authors, nurses with previous experience in collecting and analyzing qualitative data and no interpersonal relationship with the participants. *A semi-structured script developed and applied in a simulation by a team of six researchers was used, in addition to being evaluated after the first three interviews, with no need for adjustments in the questions being observed. It includes eleven questions aimed at characterizing the participant and eight guiding questions focused on the work process and the adjustments that occurred during the pandemic, such as: How do you assess the process of coping with the COVID-19 pandemic and how your work interacts with others? Which work tools are proving necessary in management of the services? How are they being accessed and incorporated? What do you perceive is being produced (changed/worked/missed) in terms of “arrangements” or ways of organizing and working, either individually or in teams?*

The study included 23 managers with a workload of 40 hours/week, linked to municipal and state public services, and working in the fight against COVID-19. They were selected for convenience, meeting the criteria of acting in the position for at least 01 month, a period understood as possible to experience the service, considering the instability in management and several manager turnovers that occurred in the period, and as representativeness of the strategic (first and second level) and operational (BHU, ECU/ECS, FA and Hospitals) levels.

Prior contact was made to present the study and schedule the interview, developed in a place suggested by the participants or by a virtual meeting via the *Google Meet* tool, recorded in digital audio or video and lasting a mean of 40 minutes. After each interview, the relevant points and the researchers’ perceptions were recorded. There was no refusal or need for repetitions.

### Data treatment and analysis

The interviews were transcribed with the aid of the *Google Docs* tool, respecting authenticity and literality. The transcribed texts were made available for knowledge and review by the interviewees, but there was no demand for review. Qualitative data saturation was confirmed by the recurrence of the contents and scope of magnitude of the codes, allowing consistent interpretations. 

In the analysis, two Thematic Coding cycles were applied, proposed by Johnny Saldaña with the aid of ATLAS.ti - The Qualitative Data Analysis Software, version 8.0, namely: 1- Structural Coding Method for initial categorization of the data *corpus*, aiming to examine the similarities, differences and relationships of the comparable segments; and 2- Theoretical Coding Method, aggregating the *corpus* by theoretical associations^13^, anchored in the concept of technical-assistance arrangements.

To increase reliability, coding was in charge of a researcher, reviewed by a second one and submitted to consensus by 3 collaborating researchers, in specific meetings for this purpose.

Triangulation between data from the interviews and documents occurred to the extent that, in the categorization process, any references to norms or guiding policy formulations that impacted on the processes of coping with the pandemic were identified, in order to better understand the bases used by the actors in the management process.

### Ethical aspects

The study met the standards of the National Health Council (*Conselho Nacional de Saúde*, CNS) for research involving human beings (Resolution No. 466/12), and obtained consent from of Manaus health authorities and approval by the Committee of Ethics in Research with Human Beings of the State University of Amazonas (*Comitê de Ética em Pesquisas/Universidade do Estado do Amazonas*, CEP/UEA) (CAAE: 31844720.6.0000.5016) and by the participants. To preserve anonymity, the statements were identified by acronyms and numbers, as shown in Figure 2.

**Figure 2:** Description of the codes used to identify the managers, considering the operation level and locus

Position	Code	Description
Initial code letters	MM	Municipal manager working in administrative headquarters
	SM	State manager acting in administrative headquarters
	H	Hospital
	U	Emergency Care Unit or Service
	BHU	Basic Health Unit
Subsequent number	Number	Sequential number corresponding to the manager of that locus
Letter M and subsequent number	M + number	Increasing number of managers participating in the study

## Results

**Figure 3:**
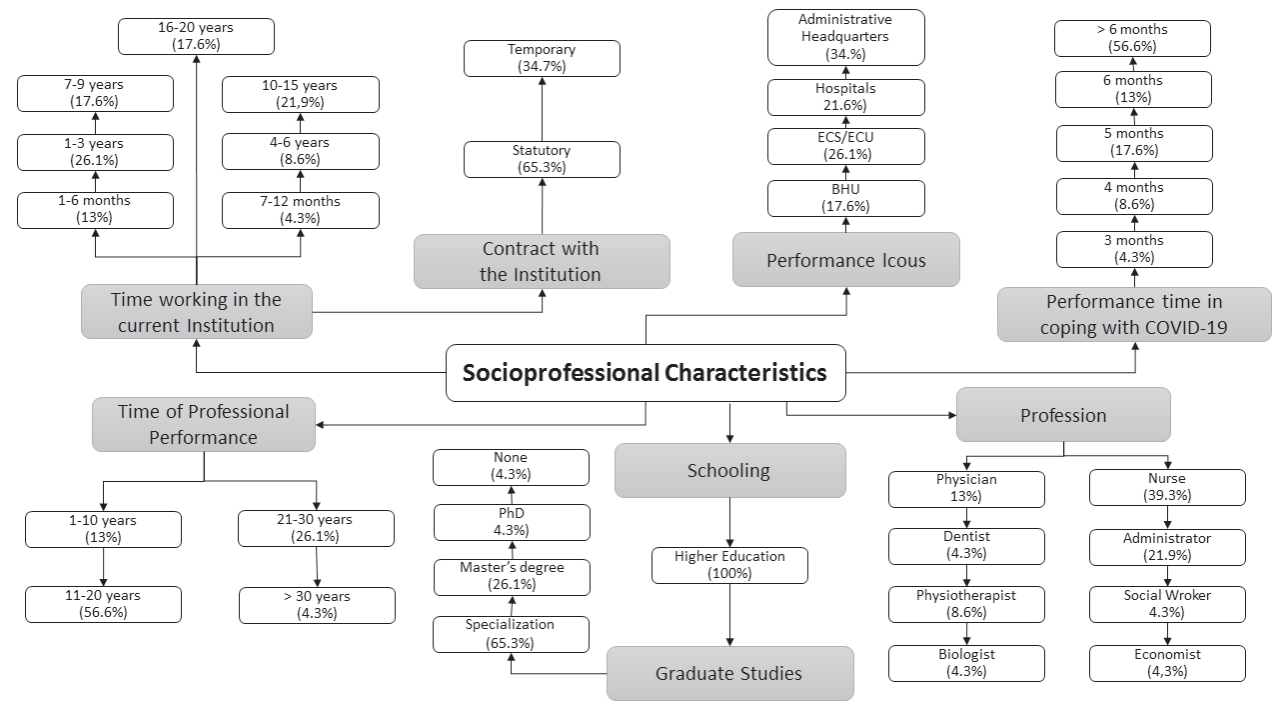
Characterization of the research participants

In relation to the participants’ profile (Figure 3), there was predominance of women (57%), 61% concentrated in the age group of 40 to 49 years old, 65% with some specialization, 39% nurses and 65% with statutory public contracts. Of these, 39% had from 16 to 20 years of training, 26% were in the institution for a period of 1 to 3 years and 57% have worked in the pandemic for a period of more than 6 months.

**Figure 4:**
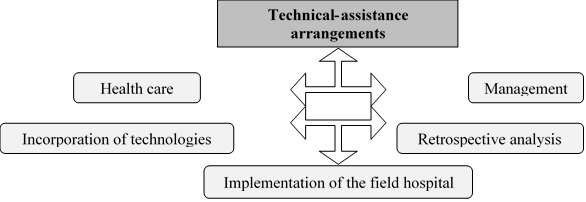
Analysis categories and magnitude

The result is divided into 05 analysis categories, with a magnitude of 238 citations, arising from the analytical process and presenting the work organization modalities, technologies and innovations incorporated in the care and management developed to face the pandemic, as shown in Figure 4.

### Technical-assistance arrangements: health care

This category addresses the arrangements designed or restructured by induction of management and health workers, aimed directly at the care of the people with suspected or confirmed COVID-19, within the scope of the point of care or the HCN. It reveals the successful experiences and reflections on weaknesses and challenges faced in terms of: reception/care flows (for suspected cases and usual demand); communication strategies with the family; monitoring of confirmed cases not hospitalized; vaccination of the aged against Influenza (drive-thru and door-to-door); team assignments for agility of response; routines for the prevention of contagion/dissemination within the service; and differentiated care to vulnerable groups.

*[...] to establish a proper flow for the patients, who at first were accumulated in the emergency service waiting for the transfer [...] we had to close sectors and transform them exclusively for COVID-19 [...] change the flow of the emergency and ward care.* (H5M23.2)

*[...] a fundamental strategy was vaccination of older adults, door-to-door. It was very hard, difficult, but we managed to keep them at their homes.* (MM6M19.59)

*When I was assisting a suspicious case, the ambulance went through a disinfection process for 40 minutes. This was one of our big anxieties, it made our response time absolutely long, due to lack of material and logistics. There weren’t so many ambulances due to lack of personnel and the ambulances were held until they were disinfected. Gowning of the team took from 20 to 30 minutes, which caused us to stop seeing many patients [...].*(MM3M4.5)

*[...] for those so-called vulnerable populations, who live on the margins, such as indigenous people, immigrants and those in street situations, adequate spaces were defined that could accommodate them in an orderly manner, offering health care, isolation and adequate care to avoid worsening [...] all this in partnership with other institutions.* (MM4M15.74)

### Technical-assistance arrangements: management

It refers to the management tools and managerial processes developed within the scope of the points of care or strategic level of the Health Secretariats, highlighting the way to organize and offer guidelines for organization of the service, aiming at accessible, resolute and timely care. The arrangements focused on four important aspects were highlighted: care coordination and operation of the services through directives [protocols, technical standards, Standard Operating Procedures (SOPs) and quality management tools]; inter-institutional cooperation; quality and agility of the information/communication; and people management in a critical scenario. As a cross-sectional element, there is the greatest challenge of management, the constant need for replanning and decision-making, involving multiple actors in a scenario of uncertainties and insufficiencies.

*We worked together with University X, we carried out care protocols based on international publications [...] the most enlightening and detailed technical notes gradually emerged, with a direct language for institutionalization in the units [...].* (SM1M2)

*[...] the Secretariat didn’t work in isolation. Another very important device was “GGIN”, a management group [...] in order to capture efforts, help and solidarity action from institutions inside and outside the municipality.* (MM4M15.14)

*We worked on the part of strategic planning, first the survey of existing and necessary hospital supplies [...] flow meters, humidifiers, oxygen points, personal protective equipment [...], and a flow analysis of this patient. At the time, we were with the LEAN project in emergencies, which helped to work on the management tools, such as 5W2H [...].* (H5M23.3)

*Daily control of how many visits we had in the SAMU, visits of flu-like syndromes in BHUs, confirmed cases, deaths and burials. [...] we have reports that are daily available.* (MM6M19.50)

Specifically about planning and decision-making, the clear impression of insufficiency is highlighted, as they make use of everything that was available. When nothing seems to be enough, there is a challenge to operating rationality and a call for creation and innovation.

*[...] acting in an integrated way, periodic meetings, involving the sectors for decision-making, and in care itself [...] communication with the directors of the units themselves, using other tools, [...] making use of everything that already existed, but then extrapolating and innovating.* (MM4M15.75)

*It is not that we were prepared for this scenario, because no one was, but we had a well-organized work process, based on the protocols, the SOPs, this made dealing with the situation a lot easier [...] we have an installed capacity and this brought an even greater overload. We reached the limit and had to start not improvising, but having the creativity to solve problems, which until then we had not, or hadn’t thought about, or hadn’t occurred in the routine.* (SM3M21.19)

### Technical-assistance arrangements: Incorporation of technologies

Regarding the technologies incorporated, aiming at the control, qualification and capillarity of the actions, development was observed both by the team, within the scope of the services, and by the managers, at a strategic level, through local and national partnerships, in order to respond to the needs of the territory in the pandemic.

The new instruments and practices, thought of as technologies that change the usual way of doing things, were shown in very simple initiatives (such as the dissemination of the “gold code” to mark cure/discharge cases), even in complex incorporations of new work processes, long held, as in the case of basic emergency care in BHUs. In summary, the reports refer to the implementation of the following changes: expanded visit; gold code; employee outpatient clinic (including virtual psychological care); integration with scientific research; specialized teleconsultation; basic urgency in BHU; training and permanent education in health by remote means; extended hours of operation for the BHU (33 Units operating from Monday to Friday from 7:00 am to 7: 00 pm); Telemonitoring; new communication channels for the user and family members (chat, telephone, email, virtual bulletin, video calls); use of new ventilatory support equipment (non-invasive ventilation mask [NIV] and Snorkel), digital technologies and applications (such as the Laboratory Surveillance System for more agile and safe dispatch of reports); virtual quarantine by applications for surveillance at airports and georeferencing of the cases.

*[...] the humanization policy acted in a fantastic way in the sense of the expanded visit, implementation of the video visit, the virtual daily bulletins... This was all a great legacy, we can work with this within the ICUs, regardless of COVID-19.* (SM1M2.86)

*It was so much code green (death), because it was 100 ICU beds! [...] A doctor sent me a message: Doctor, for God’s sake, I can’t listen to code green anymore. Can’t we get the discharge code? [...] We invented the gold code, the code of victory. Every discharge to date, whether from the ICU to the ward, or from the ward to the home, is announced by phone.*(H1M9.46)

*Having people who propose, at a time like this, to research, makes all the difference! Because they give a vision, a north [...] Research is everything and we need to have this investment! It needs to have this differentiated look.*(H1M13.26)

*[...] the pandemic forced to incorporate basic urgency in BHUs; at first there was resistance from the employees, but in the course of this process and with all the support we were giving, training, viability of inputs, the team itself gradually incorporating everything. That’s a win!* (MM4M15.54)

*[...] the management of the municipality has been working with Telemonitoring in a pioneering way. We assembled a working group for this implementation [...] and taking advantage of the situation, we’re now incorporating care focused on the chronic conditions.*(MM5M17.18)

*[..] today we have a health situation room. That makes it a lot easier. We developed Business Intelligence (BI), some access platforms, with technical languages, though light and easy to understand.* (SM3M21.10)

*I think that was the first move at the governance level! A hard technology that we developed in partnership [cites partners]. An app that the population could “download” on their cell phones, offering a set of information, in addition to accessing with the university, psychologists or even doctors. There was this device in the app [...] a group of physicians specific for teleconsultation.* (SM4M22.54)

*[...] all those who came down at the airport were required to “download” an app, we did what we call virtual quarantine [...] with the cell phone locator, we knew who these people were and if they presented any symptom [...] There was a warning button; when clicking, we triggered that person, favoring the epidemiological investigation.* (SM4M22.57)

### Implementation of a field hospital

In the process of creation and operation of field hospitals (FHs) implemented under municipal and state management, the weaknesses and strengths experienced stand out, from the perspective of structure, process and results. The reports expose the implementation and impact on society, with the following standing out: management decision-making process; strategies and reasons for implementation and deactivation; challenges faced for implementation and contribution to the HCN.

*The creation of the FH was fundamental, from the moment we had the safety that the patient leaving here had somewhere go to, that we had the protection of that life [...] the work process flowed more [...].* (BHU1M6.4)

*The FH was the accomplishment of a task, quickly, necessary, but that had an exquisite performance, because in addition to being an action paid by the city, there was the support from the private sector.* (H2M10.41)

*We had work routines created due to the need for a hospital [...] we were very careful to obey the protocols established for the patients’ needs, as well as for the care of the health professionals who were on the front line.*(H2M10.41)

*The Secretariat took the initiative to set up an FH [...] the Secretariat still does not answer for a high-complexity service, but by the political decision this was possible, with all the necessary support to set up the hospital [...] With the partners of the moment, making it happen and having the possibility and ability to effectively cure many people.* (MM4M15.48)

### Technical-assistance arrangements: retrospective analysis

The retrospective analysis of the COVID-19 coping process highlights the weaknesses and possible opportunities for improvement. It expresses the managers’ feelings and perceptions about their experiences, intensely and in the short-term. The reports gain a more personal tone, of recognition of the roles assumed, attributing meaning to keywords such as “learning”, “errors and successes”. Recognition of one’s own insufficiencies starts from the number of professionals (and their excessive workloads), passes through the very notion of contingency or catastrophe, which makes unpreparedness evident, reaching the inevitable realization that solitary work does not have the necessary reach, in addition to the lack of support from the federal government, of adherence of the population to the control measures and the marks left by the experiences.

*This issue of organization, I think it left a lot to be desired [...] They “put” COVID in all hospitals, when in reality, it was supposed to have a specific place and others for the other patients. But they spread COVID-19 in all hospitals.* (MM3M4.31)

*The SUS wasn’t prepared for this [...] the peak came and the system ended up being sucked in. We saw that there’s a lot to improve, both in the care part and in management [...] to improve the bottlenecks and gaps perceived.* (U1M7.18)

*Each professional had a contribution, they saw themselves in this process, in what they were doing and in those protocols. But, it is logical, one thing is the ideal, another thing is what is possible, and yet another thing is what is necessary. Between the ideal and the necessary, there’s a path that’s sometimes difficult to overcome, but if you have a larger organization and a cohesive team, it makes it much easier. That’s what we saw! When we needed it most, many were there beside us.* (SM3M21.24)

*[...] the management tried to do a lot, foreseeing to achieve quick social protection results, but they were not able to do it alone. I think that, to some extent, the health sector, the health management, has forgotten a very important principle: intersectorality. In the moment of panic, the management took over the problem for itself, and it should have shared this with other sectors, including the private sector. [...] the government lacked this ability to make partnerships [...] We have a very powerful industrial pole [...] this thing that the SUS can’t partner with the private sector has blocked responsiveness.* (SM4M22.30)

*The pandemic generated a superjudicialization of management [...] based on the assumption that every manager is guilty of using the resources, at a time when the law itself authorized use quickly [...] I’m not trying to justify it! The fact is that it exposes a situation on a premise that is not necessarily true, that every manager was using public resources to favor private interests to the detriment of the collective ones, and that’s not true!*(SM4M22.35)

*The relationship with the Ministry of Health (MS) was pitiful [...] if we have to count the number of deaths, many of them are due to the support that was not given by the Federal Government. [...] we saw the several changes in the ministry and this still prevailed, all and any resource release was made at least three months late [...] The federal government did not collaborate in the actions to fight against COVID-19 in Amazonas.*(SM4M22.45)

## Discussion

The reports reveal several initiatives mainly aimed at changes or adaptations in the scope of the health services in Manaus, which in isolation coincide with measures proposed in other scenarios, but that make up unique sets and only clearly noticeable in their peculiar context.

Regarding health care, the implementation of a flow of differentiated care for people with signs of flu-like syndromes occurred in state and municipal services stands out, configuring important measures to enhance interruption of the transmission chain and timely identification of symptomatic people, thus contributing to a reduction in morbidity and mortality^14^. They state strategies for the provision of care to the vulnerable population in the PHC context, reasserting the relevance of this care level in assuming the leading role in the health systems, especially in the face of public health emergencies, with emphasis on monitoring cases with territorial responsibility^15^. A number of studies developed in South Africa address the importance of PHC in coping with COVID-19, reinforcing the urgent need for investments, especially with a focus on the workers, as it directly impacts on the most resolute and timely response to the contextualized health needs, in addition to providing strengthening of the health system, with major impacts on coping with pandemics^15^ ^-^ ^16^. 

Also within the PHC scope, another action evidenced by the reports is the implementation of the basic urgency services in BHUs that occurred in April 2020, at the peak of the first crisis in the municipality, motivated by the deliberations from the instituted collegiate bodies. This measure was considered an advance for PHC, expanding the problem-solving capacity of care, alleviating the overload of the Urgency and Emergency Network (UEN), and constituting a strategic and valuable resource to face the pandemic^17^. A study carried out in Norway evidenced that people with COVID-19 demand more primary care between the first and the eighth week, resulting in recovery and in reduction of the overload of other care levels^18^. 

The managers’ attempt to assemble groups, committees and the like was noticed, in addition to the search for local, national and international partnerships, considered fundamental for strengthening and agility in decision-making, focusing on a shared management of the crisis based on the epidemiological scenario, aiming at the elaboration of guidelines for coping with the pandemic. It is a consensus that, in times of crisis, the path of dialog with experts from different areas and the participation of civil society becomes essential to promote positive impacts and avoid undesired effects of the decisions taken on health care and social protection^19^. 

The collective effort in the elaboration of SOPs, technical notes and other normative documents is notorious, intending the health services for the surveillance and timely treatment of the cases. Although the country has a robust health system, with significant capillarity, strategic planning to respond to public health emergencies is still challenging and incipient, which was not different in the municipality of Manaus, becoming the symbol of the catastrophe, due to the total collapse of the health system, with crowded hospitals and deaths at the homes due to lack of access to the services, in addition to saturated cemeteries and burials in collective graves^20^. 

In the face of chaos, the reports signal the adoption of several technical-assistance arrangements to minimize barriers to accessing the services and to enhance communication with society. The incorporation of virtual health bulletins, online care of suspected cases and monitoring of confirmed cases stood out, in addition to the use of virtual calls to promote communication with the family members of hospitalized people, based on humanization and relief for pain and suffering. Similar international initiatives showed that these innovative ways of offering care can be extended to different care levels, involving public and private institutions, amplifying access and timely care, as traditional places within the PHC scope had to close due to the unusual dynamics of COVID-19^3^. Deepening the discussion regarding the adoption of the technologies mentioned by the managers, such as Telemonitoring, automated chatbots and apps, there is an ambivalent character for public health; after all, it is indisputable that they provide access, expand resoluteness and enhance timely identification of signs of deterioration but, on the other hand, reasoning from the perspective of public ethics, equality, inclusion and social justice, the difficulties for the use by more vulnerable populations become clear, once again evidencing the social inequalities in the country^21^.

They also reveal the initiatives in setting up and structuring panels, such as the BI system, for the management of real-time information related to cases, deaths, burials, service appointments and installed capacity at the various care levels. However, there is fragmented information that does not allow a global and integrated analysis of the health services in the municipality. A comparative study of health systems in five European countries reveals that investment in data management is a valuable strategy for health surveillance measures in the COVID-19 pandemic^22^. Unfortunately, the investment in information technologies in the SUS is still insufficient, and it is imperative that the services be strengthened to provide and manage data in real time for timely surveillance, ensuring the protection, privacy, exchange and safe extraction of the diverse information generated^23^.

In the hope of amplifying health care, minimizing the collapse of the system and the harms to society, two FHs were implemented, considered emergency and temporary units, in order to respond to the health care needs at that time^24^. The first was implemented in April 2020, under municipal management, in partnership with the private sector, headquartered in a municipal school adapted for this purpose, with 43 ICU beds and 137 clinical beds exclusively for COVID-19 cases, ending its activities in June of the same year^25^.

In April 2020, the FH under state management was implemented, using the structure of an inactive private hospital, reaching 148 beds, ending activities in July 2020 and accounting for 1,800 visits^26^. Despite the relevant contribution, no record was identified in the National Registry of Health Establishments System (*Sistema de Cadastro Nacional de Estabelecimentos de Saúde*, SCNES) signaling the operation of this point of care as an FH focused on COVID-19. Closure of the FHs was understood as premature by the scientific community and society, given the severity and uncertainties inherent to the pandemic, culminating in the reactivation of the state-owned FH in January 2021, as a result of the second peak and collapse of the health system in Manaus. 

The managers recognize that the system was not prepared to face this pandemic, aggravated by the fragile performance of the MS, marked by the alternation of Ministers and positions contrary to the scientific measures recognized worldwide to control the disease, in the midst of the collapse experienced by the municipality. Coping with the pandemic is dynamic, requiring resilience, strategic planning, celerity of response, flexibility and adaptability in the use of existing resources (personnel, supplies and physical structure) from the system, aspects directly related to political leadership. In other words, government stances that simplify the seriousness of the situation and do not recognize science generate misinformation, inappropriate collective behaviors, conflicts and ineffective actions, in addition to compromising the establishment of national and international cooperation, based on best practices^15^ ^,^ ^20^.

Bold and courageous leadership is identified as a priority condition to implement innovative approaches to communication, aiming at mitigating the virus and at health care anchored to the diverse scientific evidence^27^, with the adoption of a proactive stance by managers being extremely relevant^28^, using different ways of communicating the policies adopted to society, anchored in utilitarian, republican/democratic ethical principles^29^. Brazil has taken no responsibility for accurate and adequate communication, capable of clarifying and obtaining support for scientifically-based measures, leading citizens to volatile opinions, fake news, or momentary political circumstances, contributing to the worsening of the situation.

The need to value intersectoriality was highlighted, signaled by the managers as fragile or even nonexistent, where the health sector was the protagonist in isolated actions to fight against the pandemic. The development of intersectoral actions in health is historically challenging, a collective desire to achieve, especially in times of pandemics that emanate complex decisions with major impacts on society, requiring the construction of adapted and integrated partnerships with the existing systems, consisting of multidisciplinary members, capable of execution, contextualized to social and territorial needs^30^. 

Overjudicialization is also revealed with disputes and value judgment in the face of dramatic situations, involving the value of life. The pandemic has raised questions about how scarce resources should be allocated and the complexities of balancing ethical perspectives and future realities in health care, as well as raising concerns about the legal consequences of making these decisions^31^. Judicialization of the pandemic must be viewed with caution and prudence, recognizing the health systems’ limitations worldwide. 

Although the study does not intend to perform an analysis by professional categories, the relevance and leading role of Nursing are implicit, evidenced by the greater concentration of nurses occupying management positions among the interviewees. It is noteworthy that the curricular guidelines for undergraduate Nursing studies include notions of service administration, in addition to strongly seeking the development of professional skills aimed at comprehensive care and a holistic view of health. Therefore, it is possible to assert that, among the arrangements analyzed, there is a strong presence of the Nursing category, contributing significantly to the management of services and care in pandemic times. 

The study brings about contributions to the advancement of knowledge with the potential to qualify and strengthen the public management practices in critical scenarios and the development of policies aimed at health emergencies. The following stands out for the Nursing field of knowledge: the important role of these professionals as creative leaders in the development of innovations, dynamic and responsive management modalities to the challenges of the services, the need for increasingly integrated action between different professionals and sectors; the growing demands for scientific and technological updating, based on reliable sources, as well as their role in the quality of the information produced and consumed in the work teams. In this way, the study points out fundamental elements for the permanent training of managers, for the improvement of communication between professionals, teams, management levels and with the users of the health services.

Although the study presents a potential contribution to health management in the face of public health emergencies, it is noteworthy that the reports were limited to the experiences related to the first peak of the disease that occurred in Manaus, with an urgent need for studies focused on the experiences that occurred in other scenarios and pandemic moments.

## Conclusion

The study showed that coping with the COVID-19 pandemic required dynamism and restructuring of the services to respond to the population’s needs, evidencing new technical-assistance arrangements both in terms of management and health care, with the rapid implementation of flow of differentiated care for people with signs of flu syndromes, virtual health newsletters and *online* care (telemonitoring, chatbots and apps) standing out. 

The implementation of basic urgency services in BHUs during the pandemic was evidenced, configuring an advance from the managers’ perspective, as these services were not offered in Manaus. The effort to implement field hospitals and the relevance to the peak moments of the disease in the municipality was notorious. 

Among the interviewees, the large number of nurses occupying management positions stood out, evidencing the leading role and significant contribution of the category to facing the pandemic in Manaus.

The SUS fragility was reinforced in several fields, such as information management and incipient and weak intersectoriality. The reports made it clear that the system was not prepared to face this pandemic, which was aggravated by the MS stance and behavior, from a technical and political perspective, contributing to the celerity of the collapse in Manaus.

The study presents a potential contribution to the qualification and strengthening of the public management practices in a critical scenario and the development of policies aimed at health emergencies. It is indispensable to develop other studies aimed at apprehending the experiences built during pandemics, covering not only the technical aspects, but all the subjectivity of work, even the mutual relationships between the technologies implemented and the actors’ experiences.
